# Cartilage damage and bone erosion are more prominent determinants of functional impairment in longstanding experimental arthritis than synovial inflammation

**DOI:** 10.1242/dmm.025460

**Published:** 2016-11-01

**Authors:** Silvia Hayer, Gregor Bauer, Martin Willburger, Katharina Sinn, Farideh Alasti, Roberto Plasenzotti, Tetyana Shvets, Birgit Niederreiter, Constantin Aschauer, Guenter Steiner, Bruno K. Podesser, Josef S. Smolen, Kurt Redlich

**Affiliations:** 1 Medical University of Vienna, Department of Internal Medicine III, Division of Rheumatology, Vienna 1090, Austria; 2 Medical University of Vienna, Division of Biomedical Research, Vienna 1090, Austria

**Keywords:** Functional impairment, Inflammatory joint damage, Rheumatoid arthritis animal model, TNF blockade

## Abstract

Chronic inflammation of articular joints causing bone and cartilage destruction consequently leads to functional impairment or loss of mobility in affected joints from individuals affected by rheumatoid arthritis (RA). Even successful treatment with complete resolution of synovial inflammatory processes does not lead to full reversal of joint functionality, pointing to the crucial contribution of irreversibly damaged structural components, such as bone and cartilage, to restricted joint mobility. In this context, we investigated the impact of the distinct components, including synovial inflammation, bone erosion or cartilage damage, as well as the effect of blocking tumor necrosis factor (TNF) on functional impairment in human-TNF transgenic (hTNFtg) mice, a chronic inflammatory erosive animal model of RA. We determined CatWalk-assisted gait profiles as objective quantitative measurements of functional impairment. We first determined body-weight-independent gait parameters, including maximum intensity, print length, print width and print area in wild-type mice. We observed early changes in those gait parameters in hTNFtg mice at week 5 – the first clinical signs of arthritis. Moreover, we found further gait changes during chronic disease development, indicating progressive functional impairment in hTNFtg mice. By investigating the association of gait parameters with inflammation-mediated joint pathologies at different time points of the disease course, we found a relationship between gait parameters and the extent of cartilage damage and bone erosions, but not with the extent of synovitis in this chronic model. Next, we observed a significant improvement of functional impairment upon blocking TNF, even at progressed stages of disease. However, blocking TNF did not restore full functionality owing to remaining subclinical inflammation and structural microdamage. In conclusion, CatWalk gait analysis provides a useful tool for quantitative assessment of functional impairment in inflammatory destructive arthritis. Our findings indicate that cartilage damage and bone erosion, but not synovial inflammation, are the most important determinants for progressive functional impairment in this chronic erosive arthritis model.

## Abstract (precis)

**Summary:** Gait-profile assessment is an objective quantitative measurement to monitor functional impairment in arthritis models. Longstanding functional impairment correlates with inflammation-driven structural damage.

## INTRODUCTION

Rheumatoid arthritis (RA) is a systemic and chronic inflammatory autoimmune disease characterized by synovial inflammation induction of cartilage and bone destruction. Chronic arthritis in turn leads to functional impairment and loss of mobility in affected individuals, which dramatically reduces their quality of life. Preservation of functionality is therefore the most important aim when treating RA individuals. However, different therapeutic agents might have different effects on inflammation or on the destruction of distinct structural components, such as bone or cartilage. Methotrexate, for example, is as effective as blockers of tumor necrosis factor (TNF) in reducing synovial inflammation, but less effective in blocking articular damage (Breedveld et al., 2006; Klareskog et al., 2004; Smolen et al., 2014). Recent studies in RA individuals demonstrate that even successful treatment with complete resolution of synovial inflammatory processes does not lead to a complete reversibility of joint functionality (Aletaha et al., 2006). This finding points to the crucial contribution of irreversibly damaged structural components, such as bone or cartilage, to reduced joint mobility.

Functional impairment and disability status in RA individuals is generally assessed by the health assessment questionnaire (HAQ) disability index (Aletaha, et al., 2006; Aletaha and Smolen, 2007; Fries et al., 1980). Structural joint damage can be visualized and quantified by using radiography and the Sharp scoring system or its modified versions, where the assessment of joint space narrowing (JSN), as a marker for the extent of cartilage damage, and the presence of bone erosion sites (ERO score) can be scored separately (van der Heijde, 1999). So far, studies in RA individuals that correlate functional impairment with JSN or ERO scores have demonstrated that cartilage damage could be more strongly associated than bone erosion with irreversible physical disability (Aletaha et al., 2011; Koevoets et al., 2013; Lillegraven et al., 2012; Navarro-Compan et al., 2015; Smolen et al., 2013; van der Heijde, 2011). However, those studies are limited by the fact that not all joints are affected by JSN and ERO, and that differential diagnosis of bone and cartilage erosion is sometimes difficult and might overlap.

In contrast, animal models of RA are a valuable tool to relate different structural damage components with functional disability. These models can be used to explore functional loss together with histological analysis of joint inflammation, as well as of cartilage and bone damage, in more detail. In addition, these investigations can be made at different time points during the course of the disease. Clinical signs of arthritis can be assessed by determination of paw swelling or grip strength; however, functional impairment is more difficult to determine in experimental models. In this context, assessment of gait abnormalities might offer a useful tool for the measurement of functional impairment. Previous animal studies, for example, have demonstrated gait changes in response to acute arthritis in different models (Hoffmann et al., 2010; Masocha and Parvathy, 2009; Parvathy and Masocha, 2013; Vincelette et al., 2007). However, until now, gait profiles have not been employed as a measure of functional impairment in chronic mouse models of RA. Moreover, correlation studies between gait profiles and distinct histopathological features of arthritis have also not been performed.

Human-TNF transgenic mice (hTNFtg) develop symmetrical, progressive, chronically inflamed, erosive polyarthritis starting around week 5 after birth (Hayer et al., 2007; Keffer et al., 1991). Systemic overexpression of TNF leads to the progressive development of characteristic RA features, including synovial pannus formation, infiltration of inflammatory cells, excessive development of bone-resorbing osteoclasts and concomitant formation of subchondral bone erosions, as well as cartilage damage. Both clinical and histopathological features of the disease can be prevented and reversed by blocking TNF (TNF blockade) (Redlich et al., 2004).

Therefore, in this study we sought (i) to address CatWalk-assisted gait profiles as an objective quantitative measurement of functional impairment in TNF-mediated chronic inflammatory erosive polyarthritis; (ii) to elucidate the individual impact of the different structural components (inflammation, bone erosion, cartilage damage) at different stages of the disease on functional impairment; and (iii) to investigate the reversibility of functional impairment in response to therapy.

## RESULTS

### Determination of gait parameters independent of body weight and age

We first searched for gait parameters that were independent of body weight and age. Therefore, we performed weekly gait profiles from wild-type animals from week 5 to week 15 after birth, a time period with continuous increase in body weight and size. We observed a significant longitudinal association between body weight and age and the dynamic parameter stride length, but not with the maximum or mean intensity (Table 1; see Table S1 for definitions), indicating that only stride length strongly depends on the developmental stage of growing animals and their body weight. However, we detected a significant association between age, but not body weight, and maximum and mean intensity parameters. In contrast, we observed no age- or body weight dependencies on most static parameters such as print length, print width or maximum contact area (Table 1).

**Table 1.**
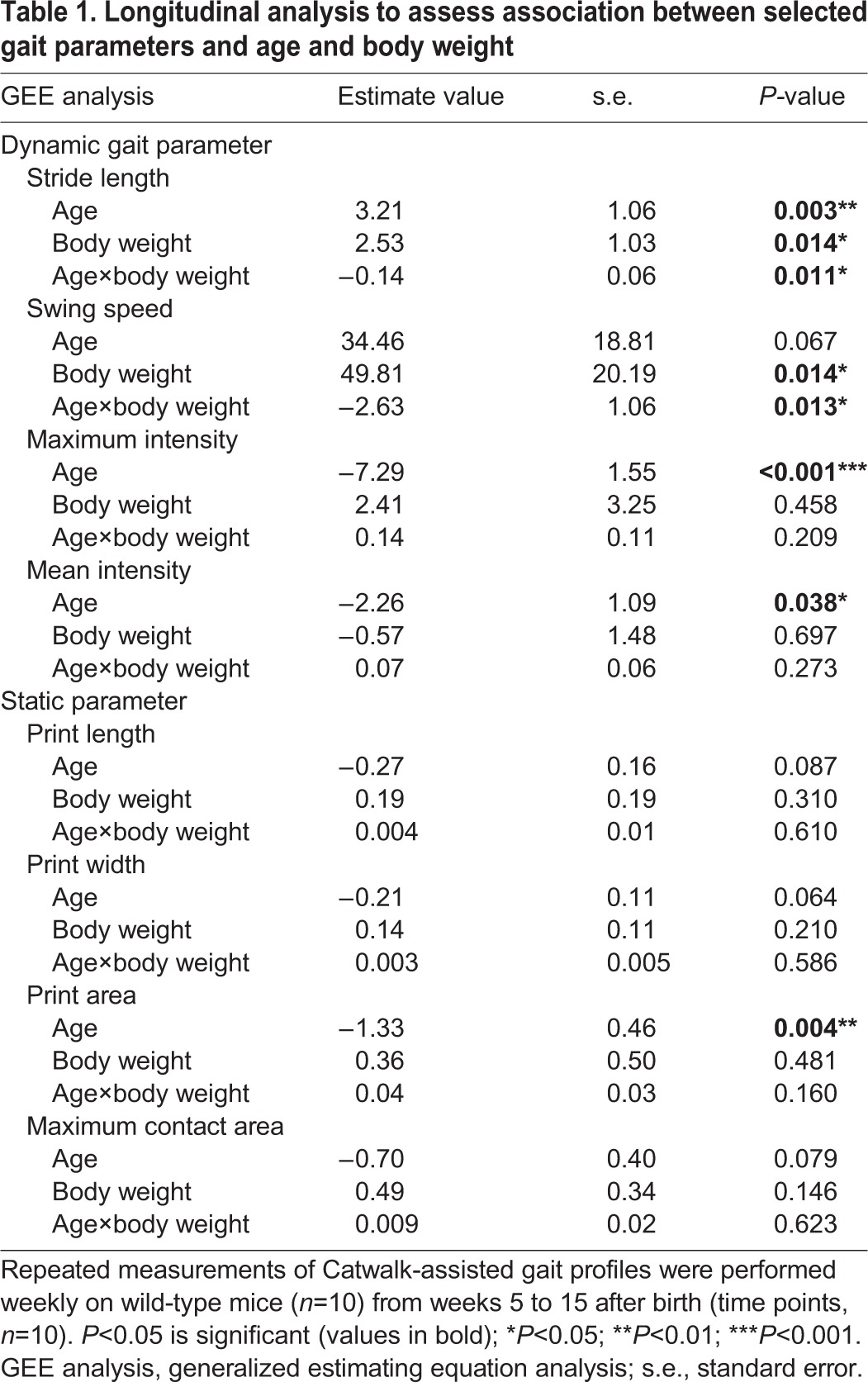
**Longitudinal analysis to assess association between selected gait parameters and age and body weight**

Taken together, we found that static parameters such as print length, print width and maximum contact area are independent of body weight and age and, thus, offer useful quantitative gait parameters to assess longitudinal gait changes and functional disabilities in hTNFtg mice affected by chronic inflammatory erosive arthritis. Age-dependent dynamic parameters, such as maximum and mean intensity and static parameter print area can be also used but only in animal studies at a certain age.

### TNF-mediated systemic inflammation causes early functional impairments

**Fig. 1.**
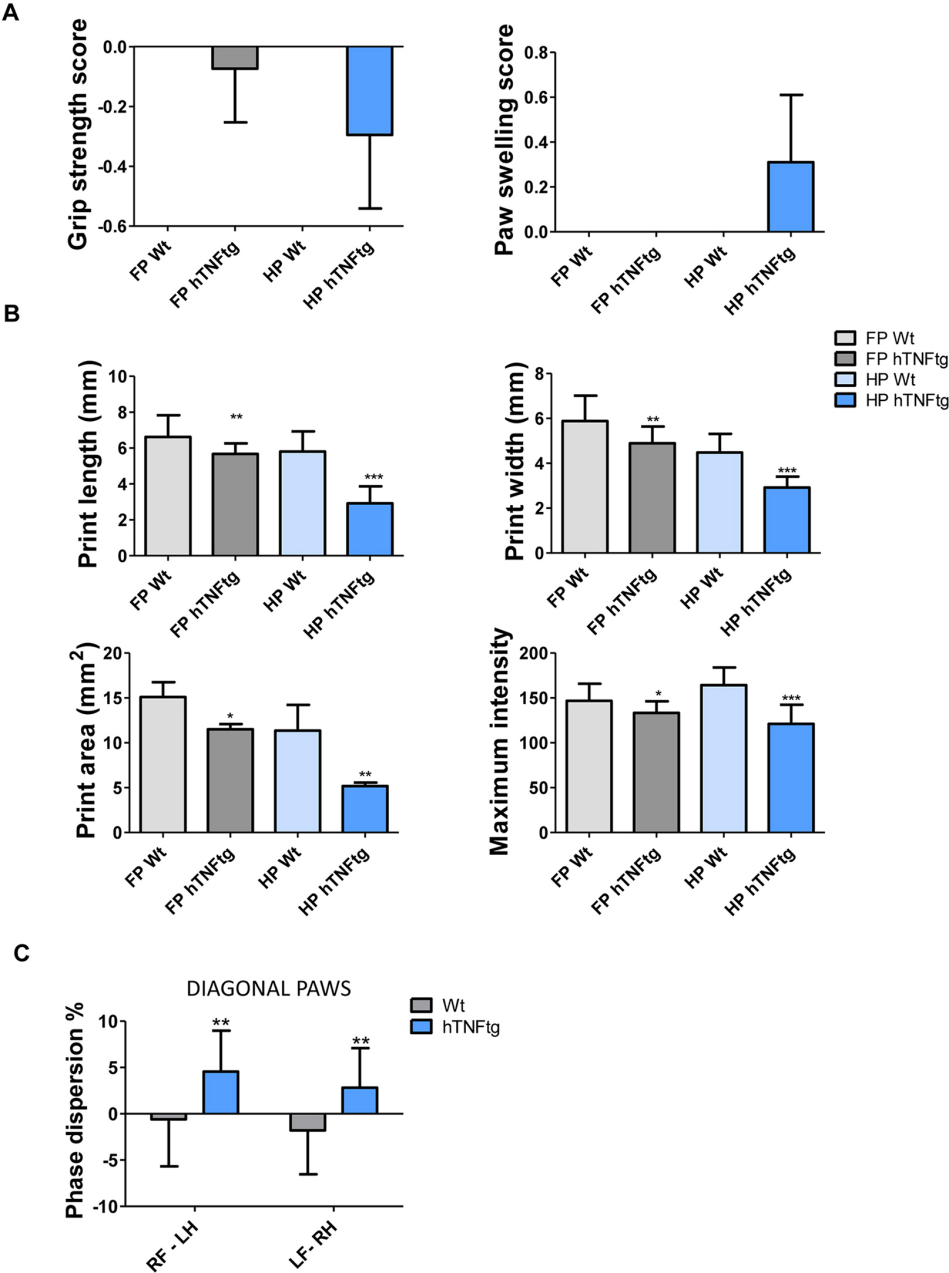
**Early functional impairment in TNF-mediated inflammatory arthritis indicated by Catwalk-assisted gait profiles from hTNFtg mice compared to wild-type animals.** (A) Clinical signs of paw swelling and decrease of grip strength in front (FP) and hind paws (HP) in 5-week-old hTNFtg mice. Wt, wild type. (B) Selected body-weight-independent gait parameters, including maximum intensity, print area, print length as well as print width in paws from hTNFtg (*n*=12) and wild-type mice (*n*=10). (C) Interlimb coordination, assessed by using phase dispersion of diagonal pairs of paws (in %). RF, right front paw; LF, left front paw; RH, right hind paw; LH, left hind paw. Data are mean±s.d. Significant differences were calculated by using unpaired Student's *t*-test (two-tailed) between hind paws or front paws from hTNFtg mice in comparison to wild-type animals; *P<0.05; **P<0.01.

To address functional impairment in inflammatory arthritis, we first evaluated gait parameters at early disease stages, when first clinical signs of arthritis such as paw swelling and reduced grip strength were apparent in hind paws from hTNFtg mice (Fig. 1A). We also observed reduced grip strength in front paws but, expectedly, no signs of macroscopically identifiable paw swelling. We found significant differences in print length, print width, print area and print maximum intensity even at week 5 after birth in hTNFtg mice in comparison to age-matched wild-type littermates, with stronger effects in hind than front paws (Fig. 1B). In contrast, we observed no differences in interlimb coordination measures, such as step pattern (using the most commonly observed alternate pattern), regularity index as well as print position between the two genotypes (data not shown). However, we found small changes in phase dispersion of the diagonal pairs of paws, which points to a slightly delayed positioning of hind paws in 5-week-old hTNFtg mice (Fig. 1C). These data indicate that functional impairment occurs even at early stages of disease and, thus, seems to be related to initial inflammatory processes.

### Progressive functional impairment of gait correlates with bone and cartilage damage rather than synovial inflammation

**Fig. 2.**
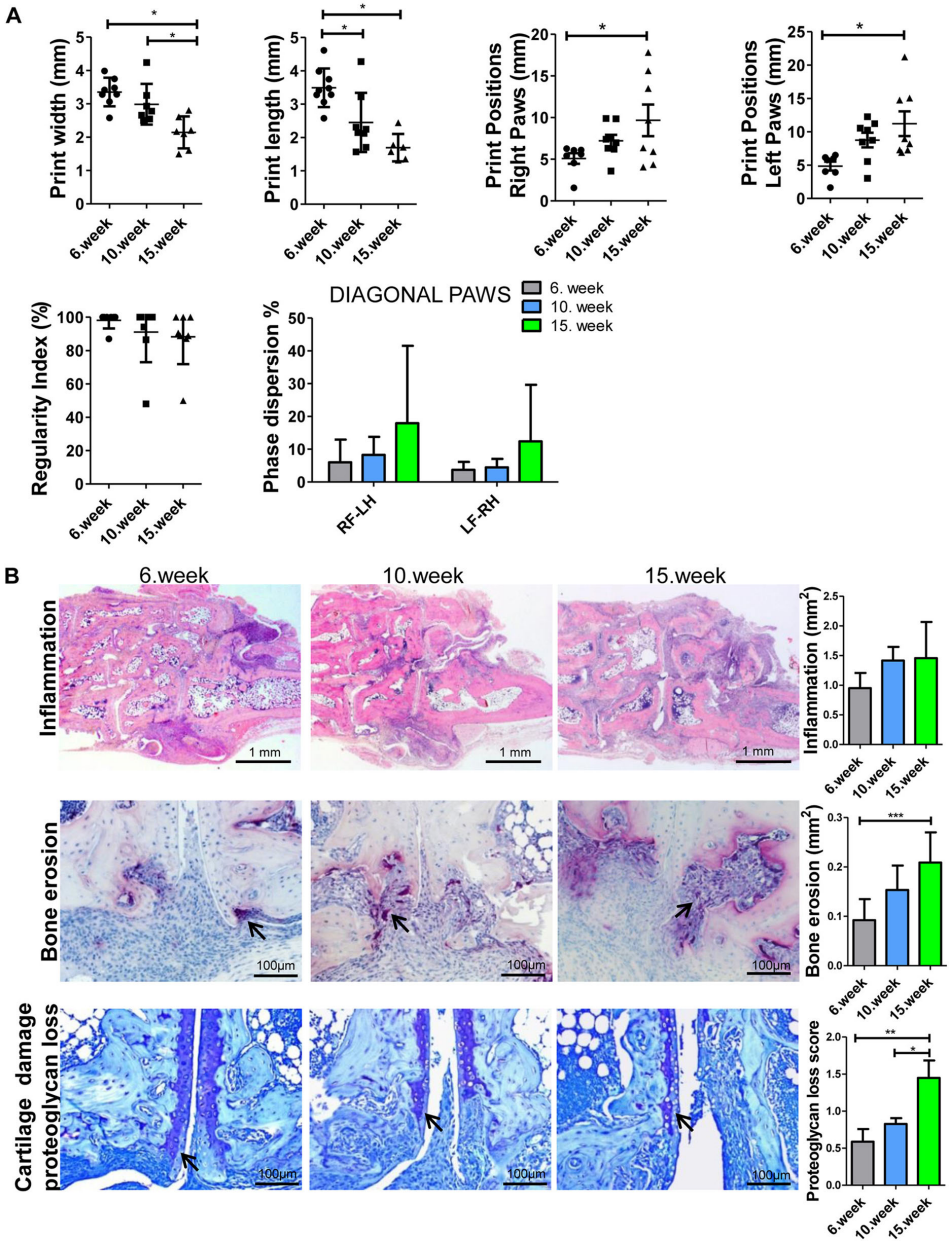
**Gait parameter changes and histopathological analysis of synovial inflammation, bone erosion and cartilage damage in hind paws from 6-, 10- and 15-week-old hTNFtg mice.** (A) Progressive decline in static gait parameters, such as print width and print length, as well as changes in interlimb coordination measures, such as print positions, regularity index and phase dispersion of diagonal paws in 6-, 10- and 15-week-old hTNFtg mice. Horizontal lines represent the mean. (B) Representative images of synovial inflammation (H&E staining), bone erosion (TRAP staining, arrows indicate purple-stained TRAP+ multinucleated osteoclasts) and cartilage damage (TB staining, arrows demonstrate destained areas indicating proteoglycan loss in articular cartilage). Original magnifications are 25× (H&E) and 200× (TRAP, TB). Right, quantitative analysis of the area of synovial inflammation (mm^2^) and bone erosion (mm^2^) as well as of proteoglycan loss of cartilage (%) assessed in hind paw sections [total number of animals, *n*=22: 6 weeks old (*n*=7), 10 weeks old (*n*=8) and 15 weeks old (*n*=7)]. RF, right front paw; LF, left front paw; RH, right hind paw; LH, left hind paw. Data are mean±s.d. *P*<0.05 is significant; *P<0.05; **P<0.01; ***P<0.001 (one-way ANOVA, Bonferroni post-test).

Next, to address the contribution of distinct joint compartments that are affected by chronic inflammation to functional impairment, we performed linear regression studies between gait parameters and different markers, including synovitis, bone erosion and cartilage damage, at different time points of the disease progression. Therefore, we addressed gait parameters independent of body weight and age in 6-, 10- and 15-week-old hTNFtg animals, and subsequently analyzed histological sections from their hind paws (7-8 animals per time point). We observed a progressive decline in print width and print length, demonstrating a significant decrease between week 6 and week 15 in both parameters, between weeks 10 and 15 of print width as well as between week 6 and week 10 of print length (Fig. 2A). Furthermore, we found a significant increase in the distance of print positions between front and hind paws as well as a continuous decrease, but without statistical significance, in the regularity index (week 6, 98.1%; week 10, 91.1%; week 15, 88.2%) and an increased phase dispersion of the diagonal paws, which indicates a progressive change in interlimb coordination (Fig. 2A).

**Fig. 3.**
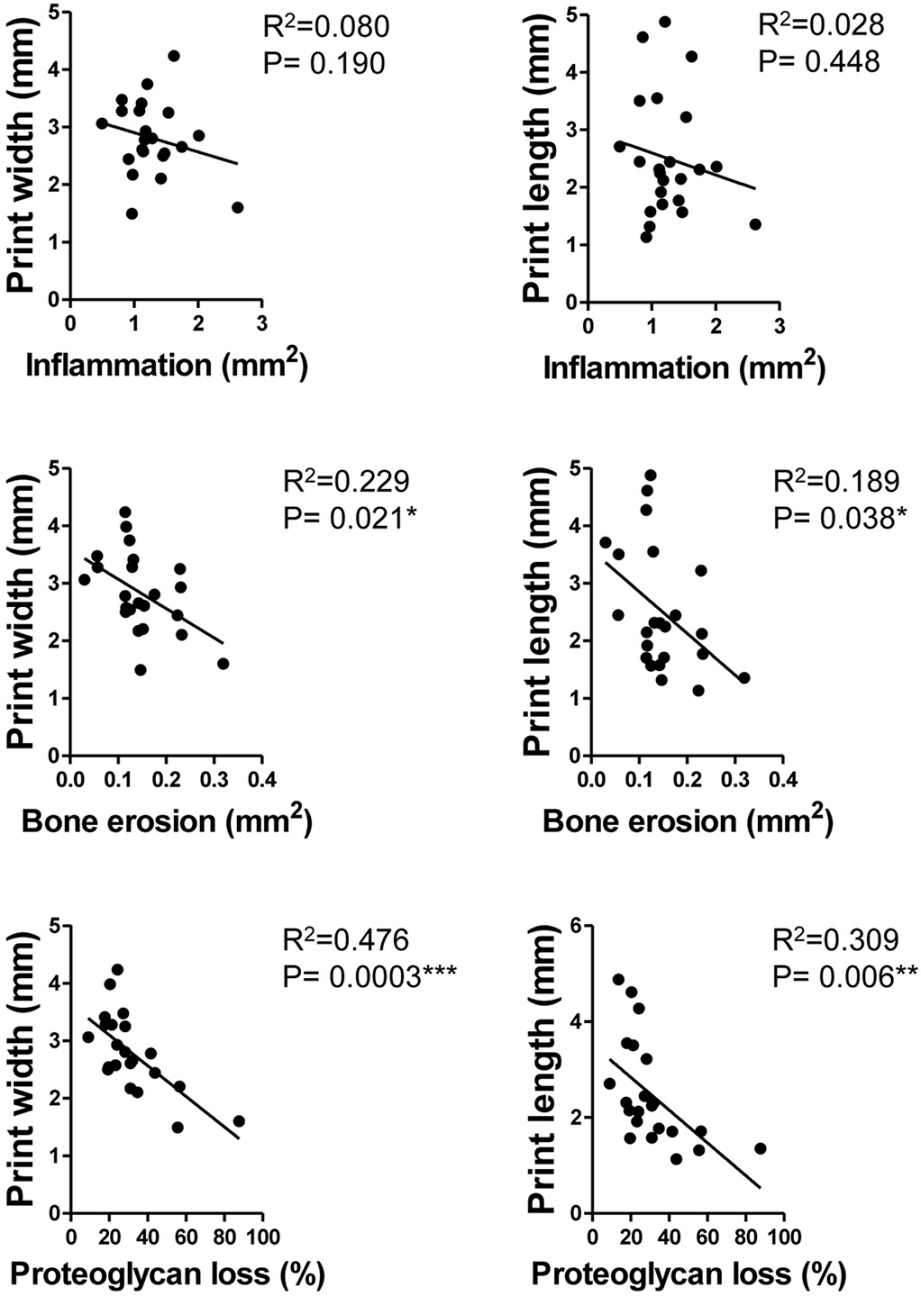
**Linear regression analysis for the association of selected gait parameters with histopathological components at different disease stages.** Gait parameters were analyzed for their association with histopathological data of synovial inflammation, bone erosion and proteoglycan loss of articular cartilage in hind paws from 6- (*n*=7), 10- (*n*=8) and 15-week-old (*n*=7) hTNFtg mice (total of 22 animals). *P*<0.05 is significant; *P<0.05; **P<0.01; ***P<0.001.

Next, we quantitatively evaluated histological sections from hind paws and found a progressive and significant increase in bone erosion, cartilage erosion and proteoglycan loss, as well as an increase in synovial inflammation; however, statistical significance was not found between values at week 6 and week 15 (Fig. 2B). Subsequently, we performed linear regression studies between those gait parameters and the abovementioned quantitatively evaluated histopathological components. Interestingly, we found a significant association of changes in distinct gait parameters, including print width and print length, with the extent of subchondral bone erosions, and this explained 18.9-22.9% of variability of gait parameter (Fig. 3). We observed an even higher significant association between proteoglycan loss of articular cartilage and those gait parameters, explaining up to 47.6% of variability. Although early gait changes strongly related to initial synovial inflammation, we observed no significant association of progressive changes in gait parameters with the extent of synovial inflammation (Fig. 3). These findings clearly indicate that functional impairment of chronically inflamed joints depends on inflammation-driven structural damage of cartilage and bone erosion.

### Blocking TNF prevents progression of functional impairment

**Fig. 4.**
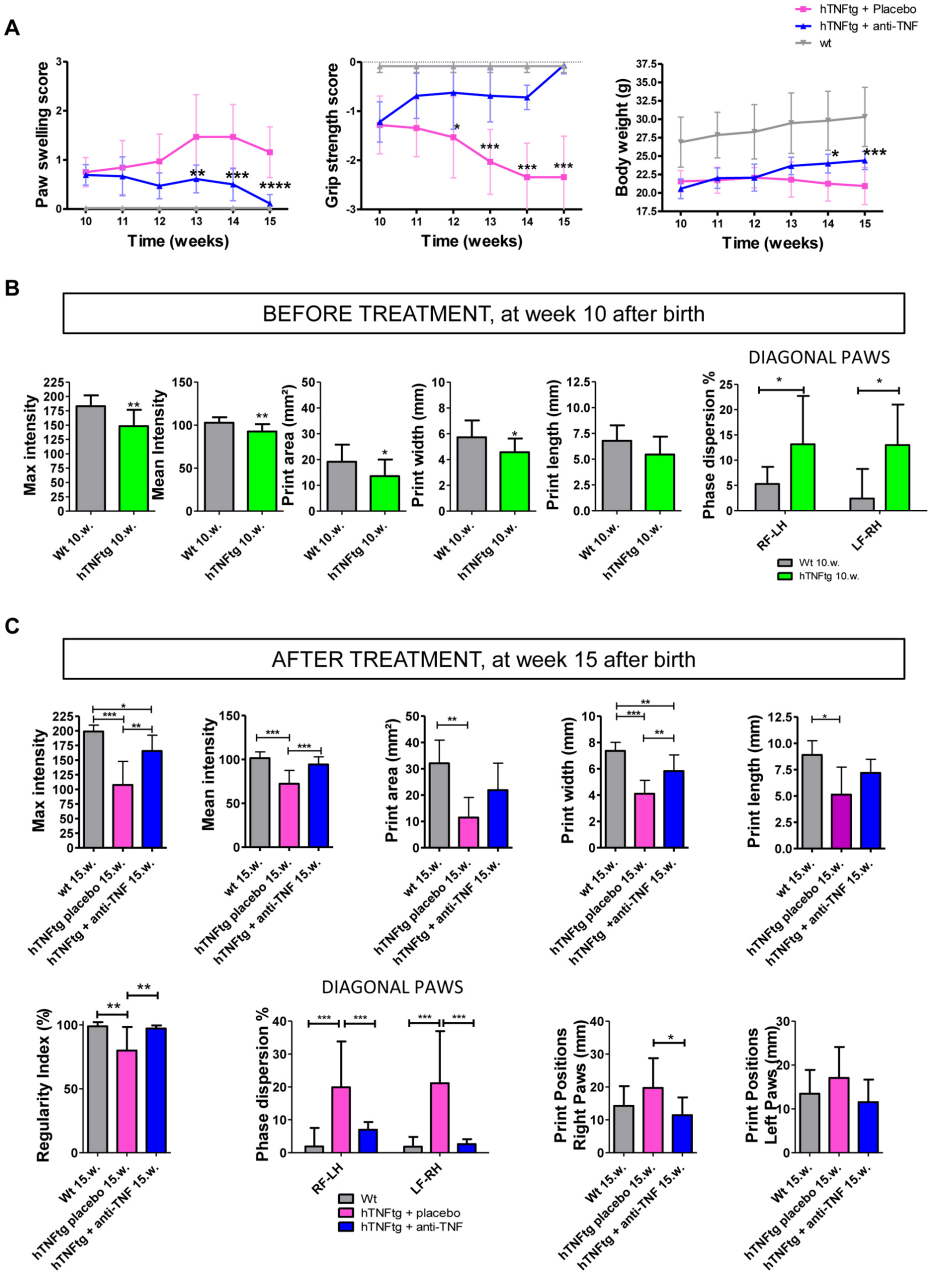
**TNF blockade reverses clinical signs of arthritis and improves functional impairment as assessed with gait analysis.** (A) Clinical course of paw swelling, grip strength and body weight in placebo- and TNF-blocker-treated (anti-TNF) hTNFtg mice and wild-type (Wt) littermates from week 10 to 15. Each group comprised nine animals. (B) Assessment of gait parameters for functional impairment in hind paws before starting treatment at week 10 (10.w.). RF, right front paw; LF, left front paw; RH, right hind paw; LH, left hind paw. (C) Gait parameters indicating functional impairment in placebo- and, partially, in TNF-blocker-treated hTNFtg mice after the treatment period at week 15. Data are mean±s.d. *P*-value<0.05 is significant; *P<0.05; **P<0.01; ***P<0.001; ****P<0.0001 (A, one-way ANOVA for repeated measures; B, two-tailed Student's *t*-test; C, one-way ANOVA, Bonferroni post-test).

In respect to the progressive decline in gait changes at late chronic disease stages and the beneficial effects of blocking TNF on both clinical and histological signs of arthritis in hTNFtg mice, we next asked whether inhibition of TNF also affects functional impairment at a progressed stage of this inflammation-driven erosive arthritis model. Therefore, we performed CatWalk gait analyses before and at the end of a 5-week treatment period with an anti-TNF drug or placebo. Treatment was started in 10-week-old hTNFtg animals, a time point at which animals already show moderate clinical signs of arthritis (Fig. 4A). Before treatment was started, significant reductions in body-weight-independent parameters, including maximum and mean intensity, print area and print width could already be detected in hTNFtg mice compared to wild-type animals, indicating marked loss of functionality at this time point (Fig. 4B). We also noticed a numerical decrease in print length but without statistical significance. Additionally, we found a significant increase in the phase dispersion of diagonal paws (Fig. 4B) but not in the regularity index or print positions (data not shown), indicating a minor impairment in interlimb coordination. After the treatment period of 5 weeks, we observed significant increases in gait parameters, including maximum intensity, mean intensity and print width in hTNFtg animals that had been treated with a TNF blocker compared to hTNFtg mice that had been treated with a placebo (Fig. 4C). Other static parameters, such as print length and print area, showed increased levels but without statistical significance between the two treatment groups. In addition, we identified a significant increase in regularity index (wild type, 99%; TNF-blocker-treated hTNFtg, 97%; placebo treated, 80%), a significant decrease in phase dispersion of diagonal paws as well as print position between TNF-blocker-treated and placebo-treated hTNFtg mice, which points to improved interlimb coordination upon treatment with a TNF blocker. To summarize, these findings indicate a beneficial effect of TNF blockade on functionality, even at late stages of chronic inflammatory erosive arthritis.

**Fig. 5.**
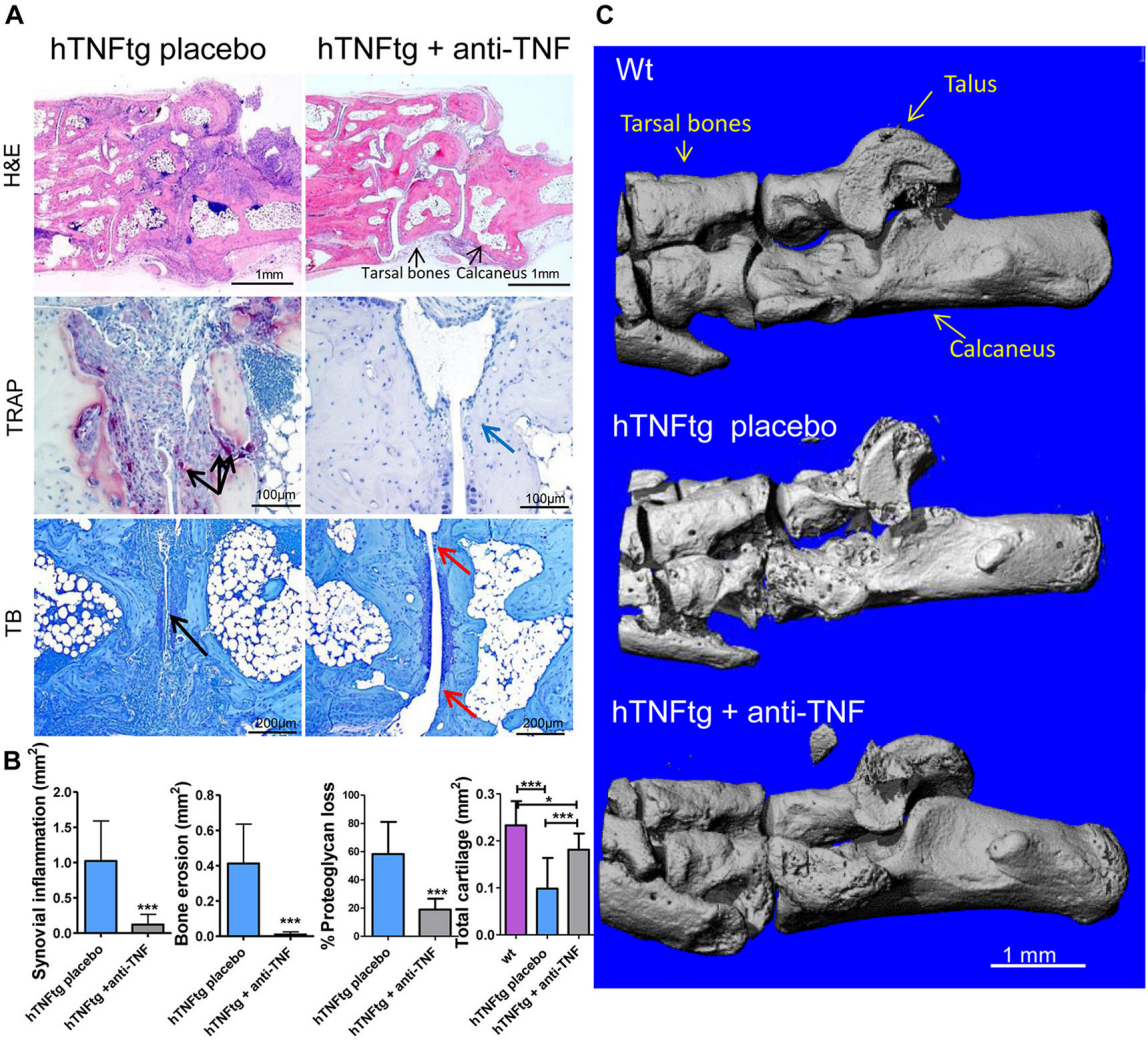
**Histopathological and microCT analysis of inflammatory joint destruction upon TNF blockade in hTNFtg mice.** (A) Representative histological paw sections of TNF-blocker (anti-TNF) or placebo-treated hTNFtg mice at week 15. Synovial inflammation was determined by H&E staining (tarsal joints), and bone erosion and osteoclasts (purple-stained cells, arrows) by using TRAP staining. Bone erosion sites were resolved after TNF blockade (blue arrow). Proteoglycan loss is illustrated by destaining of Toluidine Blue (TB) staining in the superficial cartilage layer (black arrow; magnified cuboid-calcaneal joint). Sites of irreversible residual cartilage microdamage and proteoglycan loss after TNF blockade (red arrows). (B) Quantitative analysis of the area of synovial inflammation, bone erosion and total cartilage, and the percentage of proteoglycan loss in cartilage (*n*=9 animals per group). (C) 3D microCT reconstructions from hind paw indicating the bone structure and damage. Original magnification is 25× (H&E) and 200× (TRAP, TB). Wt, wild type. Data are mean±s.d. *P*-value<0.05 is significant; *P<0.05; ***P<0.001 (two-tailed Student's *t*-test for comparison of two groups; one-way ANOVA and Bonferroni post-test for comparison of three groups).

Of note, we still found a significant difference in some gait parameters, including maximum intensity and print width in TNF-blocker-treated hTNFtg mice when compared to age-matched wild-type littermates. This indicates that functional impairment due to chronic inflammation could not be completely reversed by inhibiting TNF. However, clinical signs of arthritis, such as paw swelling and reduction of grip strength, were almost completely reversed with use of a TNF blocker (Fig. 4A). This finding points to articular changes that are not reversible upon TNF inhibition in terms of functional impairment, which cannot be determined by clinical assessment. To address the nature of these changes in more detail, we analyzed histological sections for the extent of synovitis, bone erosion and cartilage damage in hind paws. TNF blocker treatment led to a significant reduction of synovial inflammation, bone erosion and cartilage damage in the investigated tarsal joints of hind paws, compared to that seen in the placebo-treated hTNFtg mice (Fig. 5A,B). We found only very small areas of hyperplastic synovial membranes left after therapy and, thus, an almost complete histological reversal of the inflammatory synovial changes. Furthermore, we observed a complete absence of tartrate-resistant acid phosphatase (TRAP)^+^-multinucleated synovial osteoclasts, and bone erosions were virtually unseen, with only superficial and scarcely seen bony changes (Fig. 5A). Prevention of bone erosions at the ankle and the tarsal joints was also shown in three-dimensional (3D) reconstructed micro-computed tomography (microCT) images from hind paws of TNF-blocker-treated hTNFtg mice when compared to placebo-treated animals (Fig. 5C). We also found a significant reduction in cartilage damage in Toluidine-Blue (TB)-stained sections from TNF-blocker-treated animals, as represented by increased proteoglycan content and an increased area of cartilage tissue, indicating less cartilage erosion (Fig. 5A). However, we detected small areas of irreversibly damaged cartilage layers in TNF-blocker-treated hTNFtg mice. Taken together, TNF blockade markedly improved but could not fully reverse functional impairment; this might have been owing to one or more of the remaining abnormalities: subclinical synovial hyperplasia, small bone and small cartilage erosions.

## DISCUSSION

Animal models of arthritis provide insights into pathways that lead to articular inflammation and destruction, and allow semi-quantitative measurements to assess clinical changes (Joosten et al., 1999; Redlich et al., 2002; van den Berg, 2009). However, physical function is usually not assessed in these models despite the pivotal importance of maintenance or restoration of functional capacity. The reason for this shortcoming in the evaluation of experimental arthritis rests on methodological grounds given that, in man, physical function as a patient-reported outcome is assessed using questionnaires, which cannot be applied in experimental models. By contrast, impairment of physical functioning in arthritis relates, to a large extent, to impairment of mobility and strength, and this can be tested in experimental models. In the present study, we addressed the question of whether changes of gait patterns can be used as objective and quantitative measurements to assess functional impairment in an animal model of chronic inflammatory erosive polyarthritis.

### Synovial inflammation as initial event of early functional impairment

Functional impairment in RA individuals results from complex mechanisms, including synovial inflammation, bone and cartilage destruction, but the individual contribution of these processes is still under debate, especially in long-standing disease (Aletaha et al., 2011, 2006; van der Heijde et al., 2008). In our study, we detected early alterations in gait patterns (at 5 weeks after birth) and a progressive decline of distinct gait parameters at later stages of the disease. As we have previously demonstrated, at early stages of the disease, inflammation of the tendon sheets and the synovial membrane can be detected, but only marginal subchondral bone erosion and no signs of proteoglycan loss or cartilage erosion are seen (Hayer, et al., 2007). The early gait alterations indicate that even these initial inflammatory processes affect mobility and function in hTNFtg mice. This finding is in line with that of a previous study, which demonstrated gait changes even before the onset of clinical signs of arthritis in rats with Pristane-induced arthritis (Hoffmann, et al., 2010).

Also, other animal models, such as those of lipopolysaccharide (LPS)-, carrageenan- or adjuvant-induced monoarthritis support the notion that gait changes arise as a consequence of acute inflammatory processes (Angeby Moller et al., 2012; Masocha and Parvathy, 2009; Simjee et al., 2007). All these models exhibit a very rapid swelling of the paws, characterized mainly by severe subcutaneous edema accompanied by a massive infiltration of distinct inflammatory cells, synovitis, as well as expansion of resident synovial fibroblasts and macrophages. Of note, acute inflammation does not involve severe bone or cartilage erosions in these models. Therefore, initial alterations of gait parameters, such as stride length, paw pressure or paw print area have been suggested as potential measures for inflammation-mediated pain (Angeby Moller et al., 2012; Masocha and Parvathy, 2009; Parvathy and Masocha, 2013; Simjee, et al., 2007). Pain-related gait changes are highly sensitive to and can be reduced again by analgesic treatment, such as with non-steroidal anti-inflammatory drugs as well as morphine-based analgesics in the acute inflammatory stage of the disease before joint deformities are apparent (Angeby Moller et al., 2012; Heilborn et al., 2007; Simjee, et al., 2007, 2004). Of note, we could not formally exclude IgG-mediated potential analgesic effects as our control group was treated with saline. However, previous arthritis studies have demonstrated no anti-nociceptive effects of control IgG on mechanical and thermal hyperalgesia (Inglis et al., 2007).

### Progressive loss of functionality is associated with structural joint damage

In contrast to the above-mentioned induced-arthritis models, the model investigated here was not characterized by a subcutaneous edema but by chronic persistent progressive synovial inflammation, which causes the formation of massive subchondral bone erosion, proteoglycan loss and cartilage destruction, ultimately leading to complete loss of the joint architecture. Therefore, the progressive decline in gait parameters at a chronic late phase of the disease points to the influence of structural joint damage on functional impairment. Indeed, our study revealed a significant association between distinct gait parameters and bone as well as cartilage damage rather than with synovial inflammation at late stages of the disease, indicating that damage to bone and cartilage is predominantly crucial for functional impairment in hTNFtg mice. This finding is in line with a previous study describing an association of the gait parameter ‘angle between paws’ with joint destruction in the chronic phase of antigen-induced monoarthritis. However, a separate consideration of the extent of bone and cartilage erosion is lacking in that study (Boettger et al., 2009).

### Beneficial effects of TNF blockade on functionality

TNF is one of the most potent triggers of synovial inflammation, but even more so of bone and cartilage destruction in joints of RA individuals. Blockade of TNF shows strong beneficial effects in RA individuals by reducing synovitis, bone and cartilage destruction, and functional impairment (Aletaha et al., 2013; Bombardier et al., 2012; Schett et al., 2011). We and others have demonstrated the anti-inflammatory and anti-erosive capacities of TNF blockade on both local and systemic bone destruction in the hTNFtg arthritis model (Binder et al., 2013; Keffer, et al., 1991; Redlich, et al., 2004; Shealy et al., 2002; Zwerina et al., 2004). However, the effect of treatment with a TNF blocker on joint mobility and function has not been elucidated. In this study, we purposely started treatment intervention at a later stage of disease, when significantly deteriorated gait profiles were apparent. We observed beneficial effects of TNF blockade on gait parameters, which indicate not only improvement but even reversal of the functional loss caused by chronic inflammatory joint damage. However, deficits in gait parameters at this late stage of the disease could not be completely reversed through TNF blockade. At late stages of disease in this study, continued deficits in distinct gait parameters pointed to ongoing but subclinical joint inflammation or damage that could not be repaired by blocking TNF. Indeed, although we observed a significant improvement in clinical signs of arthritis, we still found marginal areas of hyperplastic synovial membranes, superficial bone erosion and small areas of cartilage erosion in the investigated tarsal joints of hind paws. Functional impairment might also be affected by irreversibly damaged tendons, but their mechanical function is difficult to prove in animal models and has therefore not been further elucidated. Our findings suggest that residual, even subclinical inflammatory processes, or irreversible small amounts of damage to bone and cartilage cause loss of function.

Taken together, we found that the assessment of gait profiles provides objective and quantitative measurements for monitoring functional impairment during the course of chronic erosive experimental arthritis. Moreover, we demonstrated a strong correlation of gait abnormalities with structural joint damage rather than with synovial inflammation at chronic late phases of the disease. Therefore, this study provides further evidence that the preservation of cartilage and bone architecture is an extremely important aspect for full functionality and should be an important consideration in the choice of therapeutic options for RA individuals.

## MATERIALS AND METHODS

### Animals

Human TNF-α transgenic mice (Tg197 strain, C57BL/6 genetic background) were originally generated by the group of George Kollias (Fleming Institute, Athens, Greece) (Keffer, et al., 1991). Mice were maintained under conventional housing conditions (humidity 50%, 22°C, 12-h light 12-h dark cycle). Heterozygous litters were genotyped by performing PCR on DNA isolated from tail biopsies as previously described (Keffer, et al., 1991). All experiments were performed in females. Age-matched non-transgenic female littermates were used as controls. All experiments were approved by the local ethical committee, Federal Ministry of Science, Research and Economics (number BMWFW66.009/0118-II/3b/2013).

### Therapeutic interventions

hTNFtg female mice received an anti-TNF antibody (infliximab; 10 mg/kg body weight) intraperitoneally three times per week, scheduled from week 10 to 15 after birth. PBS-treated hTNFtg animals served as controls. Each group comprised nine animals. After 5 weeks of treatment, animals were killed through cervical dislocation. Hind paws were isolated for subsequent histological and microCT analysis.

### Clinical assessment of arthritis

Clinical signs of arthritis, including paw swelling and grip strength, were assessed in front and hind paws using an established semi-quantitative scoring system: paw swelling from 0 to 3 (from no to severe swelling); grip strength 0 to −3 (from no to severe loss of grip strength), as described previously (Redlich, et al., 2002). In addition, body weight (g) was also assessed weekly. Clinical assessment was performed in a blinded manner by an independent investigator that was not involved in experimental treatment of the animals.

### Video-assisted gait analysis

CatWalk gait analyses (Noldus, The Netherlands) of female hTNFtg and wild-type mice were performed between 9 and 12 a.m. Mice ran spontaneously along an illuminated glass plate within a defined walkway. Using the Noldus CatWalk XT 8.1 software system, the following settings were adjusted: minimum run duration, 0.5 s; maximum run duration, 5 s; minimum number of compliant runs, 3; light intensity, 126 arbitrary units; threshold, 15 arbitrary units. Mice were allowed to spontaneously cross the walkway as often as needed to obtain three compliant runs, defined as runs with a minimum of three consecutive complete step cycles of all four paws without stopping or hesitation. All data are reported as averages of three runs. In general, a total number of 36-44 acquired steps (9- to 11-step sequences) were taken into account to determine gait parameters and step patterns. Based on the fact that hTNFtg animals develop symmetrical arthritis, gait parameters from the right and left sides of either front or hind paws were pooled for subsequent data analysis. Based on the fact that hTNFtg animals develop symmetrical arthritis, gait parameters from the right and left sides of either front or hind paws were pooled for subsequent data analysis. Numerical data of selected (i) static gait parameters, including print area, print length, print width, maximum contact area; (ii) dynamic gait parameters, including stride length, swing speed, maximum and mean intensity; and (iii) inter-limb coordination measures, such as regularity index, phase dispersion and print positions, were used (Table S1).

### MicroCT

Left hind paws were fixed in 7% formaldehyde overnight and subsequently stored in 70% ethanol at room temperature. MicroCT measurements were performed with the following settings: high resolution, 55 kVp; 145 µA, 8 W; 300 ms using a µCT35 instrument (ScancoMedical Solution). Bone samples were scanned in the presence of 70% ethanol. Structural bone damage of tarsal and ankle joints was revealed by 3D reconstruction images.

### Quantitative histopathological analysis

Right hind paws from hTNFtg and wild-type animals were fixed in 7% formaldehyde for 6 h, followed by decalcification in 14% EDTA buffer (pH 7.2) for 4 to 6 days. Decalcified 2-μm serial sections were stained with hematoxylin & eosin (H&E) for assessment of synovial inflammation, for TRAP (leukocyte staining kit; Sigma, St. Louis, MO) to determine severity of bone erosions and the number of osteoclasts, and with Toluidine Blue (TB) for evaluation of cartilage proteoglycan loss. Histological assessment was performed in a blinded manner by an independent investigator that was not involved in experimental treatment of the mice. Quantitative analysis of synovial inflammation, cartilage damage and bone erosion in the tarsal area was assessed with an Axioskop 2 microscope (Carl Zeiss Vision GmbH, Germany) and the Osteomeasure software (OsteoMetrics, Decatur, GA), as described previously (Redlich, et al., 2002; Zwerina, et al., 2004). In more detail, the area of synovial inflammation, bone erosion and total cartilage tissue (indicates cartilage erosion) was manually drawn in the tarsal joints, including the calcaneocuboid, cuneonavicular, intercuneiform to tarsometatarsal joints (mean from at least two serial sections per animal). Corresponding quantitative data are given in mm^2^. The number of osteoclasts was determined in the same joints in TRAP-stained sections by counting synovial TRAP+ multinucleated osteoclasts localized in bone erosions and adjacent synovial tissue. The second determinant for cartilage breakdown, proteoglycan loss, was evaluated based on the extent of destaining of Toluidine Blue in non-mineralized cartilage layer using an arbitrary scoring system, ranging from 0 (normal fully stained cartilage layer) to 3 (fully destained cartilage layer) (Koenders et al., 2005; Lubberts et al., 2003).

### Correlation studies and statistical analysis

Statistical analyses and graphs were made using GraphPad Prism software. Data are expressed as means± s.d. All data were consistent with a normal distribution according to a Kolmogorov–Smirnov test. Gait parameters were compared between two animal groups at a defined time point using a two-tailed Student's *t*-test, and between more than two animal groups using one-way ANOVA and Bonferroni post test. Progression within the groups was calculated with one-way ANOVA for repeated measures and Bonferroni post test. Linear regression analyses were performed to associate gait parameters with histopathological markers. The longitudinal relationship between gait parameters and age and body weight were assessed from repeated measures in the same wild-type animals by using generalized estimating equation (GEE) analysis (using SAS 9.4 software, PROC Genmod). *P*-values of <0.05 were considered as statistically significant.
